# Global Profiling of Metabolic Adaptation to Hypoxic Stress in Human Glioblastoma Cells

**DOI:** 10.1371/journal.pone.0116740

**Published:** 2015-01-29

**Authors:** Paulina Kucharzewska, Helena C. Christianson, Mattias Belting

**Affiliations:** 1 Department of Clinical Sciences, Section of Oncology and Pathology, Lund University, Lund, Sweden; 2 Skåne Oncology Clinic, Skåne University Hospital, Lund, Sweden; University of Patras, GREECE

## Abstract

Oncogenetic events and unique phenomena of the tumor microenvironment together induce adaptive metabolic responses that may offer new diagnostic tools and therapeutic targets of cancer. Hypoxia, or low oxygen tension, represents a well-established and universal feature of the tumor microenvironment and has been linked to increased tumor aggressiveness as well as resistance to conventional oncological treatments. Previous studies have provided important insights into hypoxia induced changes of the transcriptome and proteome; however, how this translates into changes at the metabolite level remains to be defined. Here, we have investigated dynamic, time-dependent effects of hypoxia on the cancer cell metabolome across all families of macromolecules, *i.e*., carbohydrate, protein, lipid and nucleic acid, in human glioblastoma cells. Using GC/MS and LC/MS/MS, 345 and 126 metabolites were identified and quantified in cells and corresponding media, respectively, at short (6 h), intermediate (24 h), and prolonged (48 h) incubation at normoxic or hypoxic (1% O_2_) conditions. In conjunction, we performed gene array studies with hypoxic and normoxic cells following short and prolonged incubation. We found that levels of several key metabolites varied with the duration of hypoxic stress. In some cases, metabolic changes corresponded with hypoxic regulation of key pathways at the transcriptional level. Our results provide new insights into the metabolic response of glioblastoma cells to hypoxia, which should stimulate further work aimed at targeting cancer cell adaptive mechanisms to microenvironmental stress.

## Introduction

Glioblastoma (GBM) constitutes the most common type of primary tumors of the brain, and is characterized by severe hypoxia, vascular hyperproliferation and therapy resistance. With combined extensive surgery, radiochemotherapy and adjuvant chemotherapy the median overall survival of GBM patients is only approx. 15 months [1]. Hypoxia is primarily a pathophysiologic consequence of uncontrolled tumor growth, resulting in structurally and functionally disturbed microcirculation [2, 3]. It is a major driver of cancer progression as it provides a strong selective pressure resulting in the survival and propagation of the most aggressive malignant cells. The magnitude of hypoxia has been associated with invasion, metastasis, tumor recurrence, decreased patient survival and intrinsic resistance to chemoradiotherapy. Thus, adaptive mechanisms to hypoxic stress are potential treatment targets of GBM and other tumor types; however, an increased understanding of such mechanisms is critical for the development and implementation of hypoxia-targeted interventions.

Hypoxic tumor cells undergo a significant shift from oxidative phosphorylation in mitochondria towards anaerobic glycolysis, manifested by *e.g*. induction of glucose transporters (GLUTs), glycolytic enzymes and lactate dehydrogenase (LDH) [4]. Overexpression of pyruvate dehydrogenase kinase 1 (PDK1) [5, 6] inactivates pyruvate dehydrogenase (PDH), resulting in efficient down regulation of the tricarboxylic acid (TCA) cycle and oxidative phosphorylation. Along with alterations of bioenergetics, hypoxic tumor cells may show increased synthesis of glycogen [7], lipids [8] and phosphorylated lipid metabolites [9].

Comprehensive metabolomics approaches that simultaneously detect changes in a variety of metabolites in cells from different oxygenation conditions should be critical for the identification of potential metabolic ‘Achilles’ heels’ of cancer cells [10]. In this study, we have used metabolomics and gene expression profiling approaches to investigate the dynamics of metabolic responses to hypoxic stress in human GBM cells.

## Materials and Methods

### Hypoxic treatment and sample collection

U-87 MG cells (HTB-14; purchased from American Type Culture Collection) were incubated in DMEM supplemented with 1% bovine serum albumin (BSA), 2 mM L-glutamine, 100 U/ml penicillin and 100 μg/ml streptomycin for 6, 24 and 48 h at normoxic (21% O_2_) or hypoxic conditions (1% O_2_) in an InVivo_2_ Hypoxia Work station 400 (Ruskinn Technology Ltd). Conditioned media were collected and centrifuged at 800 x g for 3 min to eliminate cell debris. Supernatant fractions (500 μl) transferred to 2 ml cryovial tubes were flash-frozen in liquid nitrogen. Cells were collected by trypsinization, centrifuged at 800 x g for 3 min, counted, transferred to 2 ml cryovial tubes and flash-frozen in liquid nitrogen. Both media and cells were stored at -80°C until time of analysis.

### Sample preparation for metabolic profiling

Six independent preparations of normoxic and hypoxic U-87 MG cells and corresponding conditioned media were prepared from each time-point for metabolic analyses using a non-targeted platform that enables relative quantitative analysis of a broad spectrum of metabolites [11, 12]. The metabolic profiling analysis was based on three independent platforms (Metabolon Inc.); ultrahigh performance liquid chromatography/tandem mass spectrometry (UHPLC/MS-MS2) in the positive ion mode, UHPLC/MS-MS2 in the negative ion mode, and gas chromatography/mass spectrometry (GC/MS). Samples were processed essentially as described previously [11, 12]. Briefly, on the day of extraction, samples were thawed on ice, proteins were precipitated with methanol using an automated MicroLab STAR system (Hamilton Company). Recovery standards were added prior to the extraction process for monitoring of the extraction efficiency. The resulting extract was divided into two fractions for UHPLC/MS and GC/MS analysis, respectively, and placed briefly on a TurboVap (Zymark) to remove the organic solvent, followed by freeze drying under vacuum. For UHPLC/MS analysis, extract aliquots were reconstituted in either 0.1% formic acid for positive ion UHPLC/MS, or 6.5 mM ammonium bicarbonate pH 8.0 for negative ion UHPLC/MS. Reconstitution solvents contained instrument internal standards that were used to monitor instrument performance and as retention index markers. For GC/MS analysis, aliquots were derivatized under dried nitrogen using bistrimethyl-silyl-triflouroacetamide (BSTFA). The derivatization mixture contained a series of alkyl benzenes for use as retention time markers.

### Ultrahigh performance liquid chromatography/Mass Spectrometry (UHPLC/MS)

UHPLC/MS was carried out using a Waters Acquity UHPLC (Waters Corporation) coupled to an LTQ mass spectrometer (Thermo Fisher Scientific Inc.) equipped with an electrospray ionization (ESI) source and linear ion-trap (LIT) mass analyzer. Two separate UHPLC/MS injections were performed on each sample using separate dedicated columns: one optimized for positive ions and one for negative ions. The mobile phase for positive ion analysis was 0.1% formic acid in H_2_O (solvent A) and 0.1% formic acid in methanol (solvent B), while the mobile phase for negative ion analysis consisted of 6.5 mM ammonium bicarbonate, pH 8.0 (solvent A) and 6.5 mM ammonium bicarbonate in methanol (solvent B). The acidic extracts were monitored for positive ions and the basic extracts for negative ions in independent injections. The extracts were loaded via an autosampler (Waters Acquity), and gradient eluted (0% B to 98% B over 11 min) directly into the mass spectrometer at a flow rate of 350 μl/min. The MS analysis alternated between MS (99–1000 m/z) and data-dependent MS2 scans using dynamic exclusion.

### Gas chromatography/Mass Spectrometry (GC/MS)

The derivatized samples for GC/MS were separated on a 5% phenyldimethyl silicone column with helium as the carrier gas and a temperature ramp from 40°C to 300°C over a 16 min period, and then analyzed on a Thermo-Finnigan Trace DSQ MS (Thermo Fisher Scientific, Inc.) operated at unit mass resolving power with electron impact ionization and a 50 to 750 atomic mass unit scan range. The instrument was tuned and calibrated for mass resolution and mass accuracy on a daily basis.

### Data Extraction

Data extraction of the raw mass spectra data files was loaded into a relational database and manipulated without resorting to BLOB manipulation. Once in the database the information was examined and appropriate QC limits were imposed. Peaks were identified using Metabolon’s proprietary peak integration software, and component parts were stored in a separate and specifically designed complex data structure.

### Compound identification

Metabolites were identified by automated comparison and spectra fitting to a chemical standard library, as previously described [11, 12]. Identification of known chemical entities was based on comparison with metabolomic library entries of purified standards. To date, >1000 commercially available purified standard compounds have been registered into LIMS. The combination of chromatographic properties and mass spectra provides an indication of a match to the specific compound or an isobaric entity.

### Statistical analysis of data from metabolomic profiling

For statistical analysis and data display purposes, any missing values were assumed to be below the limits of detection, and these values were imputed with the compound minimum (minimum value imputation). Statistical analysis of log-transformed data was conducted using “R” (http://www.r-project.org). Welch’s two-sample t-tests were conducted to compare data between experimental groups. Multiple comparisons were accounted for by estimating the false discovery rate (FDR) using q values [13].

### Gene expression microarray analysis

Total RNA was extracted with TRIzol Reagent (Life Technologies) and quantified using a Nanodrop ND-1000 spectrophotometer (Saven Werner). RNA integrity was verified on an Agilent 2100 Bioanalyzer. Microarray experiments were performed at SCIBLU Genomics at Lund University, Sweden using Illumina HumanHT-12 v3 Expression BeadChip. Three independent preparations of normoxic and hypoxic U-87 MG cells were analyzed on the BeadChip. Data filtration and normalization were performed using BASE2, and subsequent analyses on transcripts showing a detection P-value below 0.01 were performed using the R statistical programming environment (http://www.r-project.org). Hypoxia-mediated gene expression changes, calculated as the ratio of mean hypoxia intensity divided by mean normoxia intensity across the triplicate assays, were determined. The MeV software was used to extract transcripts differentially expressed between normoxic and hypoxic cells. The data have been deposited in the Gene Expression Omnibus (GEO) database, http://www.ncbi.nlm.nih.gov/geo (accession no. GSE45301).

## Results and Discussion

### Hypoxic regulation of the metabolome of glioblastoma cells and media

**Fig 1:**
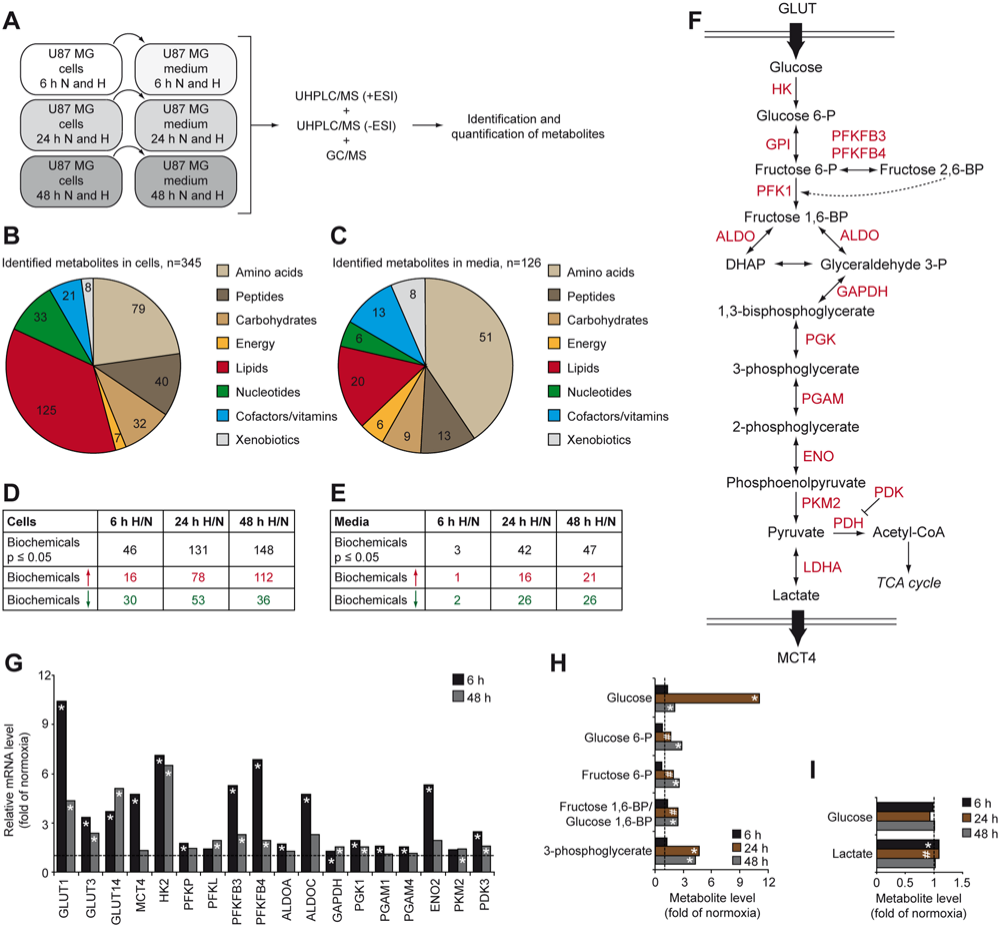
The metabolic phenotype of hypoxic glioblastoma cells. A, U87 MG cells were grown at normoxic (N, 21% O_2_) or hypoxic (H, 1% O_2_) conditions for 6, 24 or 48 h. Cells (n = 6 for each time point) and the corresponding conditioned media (n = 6 for each time point) were analyzed on three separate mass spectrometry platforms. The identification of known chemical entities was based on comparison to metabolic library entries of purified standards. B and C, Class distribution of identified metabolites in cells (B) and conditioned media (C). D and E, Summary of number of biochemicals that was significantly down- or up-regulated in hypoxic cells (D) and corresponding conditioned media (E) when compared with normoxic conditions. F, Schematic diagram of glycolysis. G, Hypoxia-driven transcriptional activation of genes encoding proteins involved in glycolysis at the indicated time-points. Data represent fold change of mRNA levels in hypoxic *vs*. normoxic cells. H and I, Hypoxic modulation of glycolysis associated metabolites. Data represent fold change of metabolite levels in hypoxic GBM cells (H) and their conditioned media (I) compared with normoxic samples. * P < 0.05; # 0.05 < P < 0.1. ALDO, aldolase; ENO2, enolase 2; GPI, glucose-6-phosphate isomerase; HK, hexokinase; LDHA, lactate dehydrogenase A; PDK3, pyruvate dehydrogenase kinase 3; PFK, phosphofructokinase; PFKFB, 6-phosphofructo-2-kinase/fructose-2,6-biphosphatase; PGAM, phosphoglycerate mutase; PGK1, phosphoglycerate kinase 1; PKM2, pyruvate kinase isoenzyme type-M2.

We studied the effects on the metabolite profile of U-87 MG GBM cells and corresponding media at short (6 h), intermediate (24 h), and prolonged (48 h) periods of hypoxia. Metabolome analyses were performed in a non-targeted fashion using UHPLC/MS (+ESI), UHPLC/MS (-ESI), and GS/MS mass spectrometry platforms (Fig. 1A). We identified a total of 345 metabolites in cells, and 126 metabolites in conditioned media (Fig. 1B–E). Quantitative analysis of compounds up- or down-regulated in hypoxic compared with normoxic GBM cells indicated that significant metabolic changes were mainly observed at prolonged exposure to hypoxia (Fig. 1D and S1 Table). The same time dependence was observed when comparing conditioned media from normoxic and hypoxic GBM cells (Fig. 1E and S2 Table). There was a strong overweight of metabolite accumulation in hypoxic cells over time, whereas changes in corresponding media were more evenly distributed between increased and decreased metabolite levels (*cf*. Fig. 1D and E).

### Hypoxia augments the Warburg effect in glioblastoma cells

Cancer cells take up higher amounts of glucose and exhibit a relative increase of glycolysis and lactic acid fermentation and decrease of oxidative phosphorylation to generate metabolic energy. This phenotype is known as the Warburg effect and is a hallmark of cancer cell metabolism [14]. Our gene expression analyses of GBM cells could confirm previous observations on hypoxic enhancement of this metabolic phenotype with increased levels of transcripts encoding proteins involved in glucose uptake and glycolysis (Fig. 1F and G). In most cases this response was transient, *i.e*. the effect was relatively greater at short as compared with prolonged hypoxic treatment (Fig. 1G). In accordance with the transcriptomic data, hypoxic GBM cells exhibited increased levels of glucose (approx. 11-fold) and several glycolysis intermediates (Fig. 1H). However, this was not reflected by increased glucose consumption from media as compared with time-matched normoxic cells (Fig. 1I), conceivably due to the great excess of extracellular glucose. At hypoxia, HIF1α diminishes pyruvate flux into the TCA cycle by PDK-mediated inhibition of PDH [5, 6]. Accumulating pyruvate becomes converted into lactate by LDH, and then translocated to the extracellular space by monocarboxylate transporter 4 (MCT4) [15]. This step is crucial for regeneration of NAD^+^ from NADH for use in the glycolysis. Gene expression data showed that hypoxia induces a transient increase of PDK3 and MCT4 (Fig. 1G), and metabolite analyses showed significantly increased lactate in hypoxic compared with normoxic GBM cells (Fig. 1I).

### Glucose flux into non-glycolytic metabolic pathways in hypoxic GBM cells

**Fig 2:**
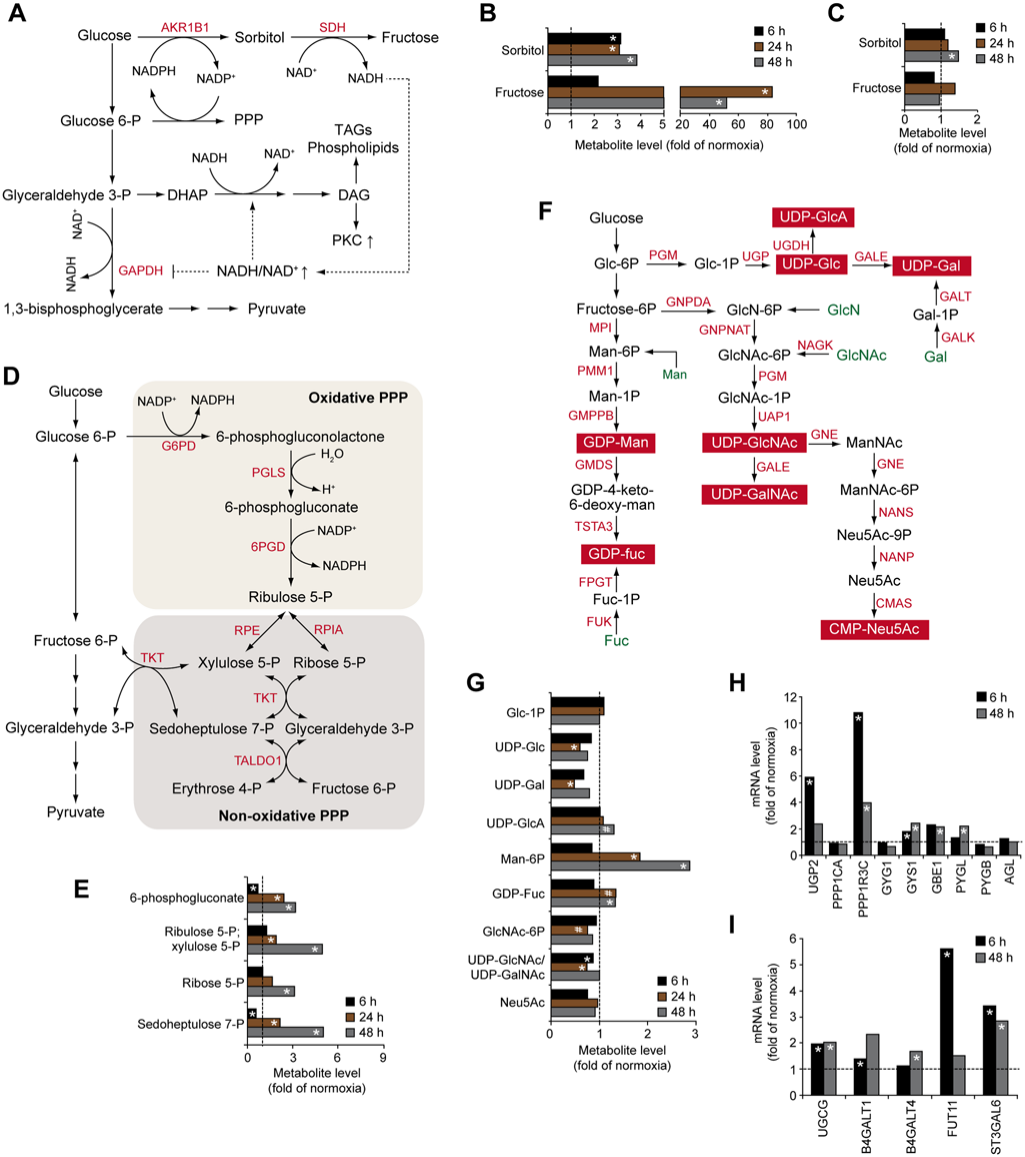
Altered glucose shunting in hypoxic GBM cells. A, Schematic of the polyol pathway. B and C, Hypoxic effects on metabolites of the polyol pathway in cells (B) and media (C). D, Schematic of the pentose phosphate pathway (PPP). E, Hypoxic modulation of PPP metabolites in cells. F, Schematic of metabolic pathways involved in nucleotide sugar synthesis. G, Hypoxic modulation of nucleotide sugars and metabolites involved in their synthesis. H and I, Hypoxic induction of mRNAs encoding enzymes involved in glycogen synthesis (H) and protein and lipid glycosylation (I) at the indicated time-points. Data represent fold change in hypoxic compared with normoxic cells. * P < 0.05; # 0.05 < P < 0.1. CMAS, cytidine monophosphate N-acetylneuraminic acid synthetase; FPGT, fucose-1-phosphate guanylyltransferase; FUK, fucose kinase; GALE, UDP-galactose-4 epimerase; GALK, galactokinase; GALT, galactose-1-phosphate-uridylyltransferase; GMDS, GDP-mannose 4,6-dehydratase; GMPPB, GDP-mannose pyrophosphorylase B; GNE, glucosamine (UDP-N-Acetyl)-2-epimerase/N-acetylmannosamine kinase; GNPDA, glucosamine-6-phosphate isomerase; GNPNAT, glucosamine-phosphate N-acetyltransferase 1; G6PD, glucose-6-phosphate dehydrogenase; MPI, mannose phosphate isomerase; NAGK, N-acetylglucosamine kinase; NANP, N-acetylneuraminic acid phosphatase; NANS, N-acetylneuraminic acid synthase; 6PGD, phosphogluconate dehydrogenase; PGLS, 6-phosphogluconolactonase; PGM, phosphoglucomutase; PMM1, phosphomannomutase 1; RPE, ribulose-phosphate 3-epimerase; RPIA, ribose-5-phosphate isomerase; TALDO1, transaldolase 1; TKT, transketolase; TSTA3, GDP-L-fucose synthetase; UAP1, UDP-N-acteylglucosamine pyrophosphorylase 1; UGDH, UDP-glucose 6-dehydrogenase; UGP, UDP—lucose pyrophosphorylase.

Apart from being the primary source of energy in hypoxic cells, glucose and glycolysis intermediates are substrates for the synthesis of cellular macromolecules (S1 Fig.) [14]. We found that hypoxia substantially induced the levels of both sorbitol (approx. 3-fold) and fructose (up to > 80-fold) (Fig. 2A and B), indicating the profound activation of the alternative glucose metabolism route, the polyol pathway, in hypoxic glioma cells. The polyol pathway, which is induced by hyperglycemia and implicated in diabetic retinopathies [16], is a two-step metabolic pathway in which aldose reductase (AKR1B1) reduces glucose to sorbitol while its cofactor NADPH is oxidized to form NADP^+^. Sorbitol is then oxidized to fructose by sorbitol dehydrogenase (SDH) with the concomitant reduction of NAD^+^ to NADH (Fig. 2A). Induction of aldose reductase may rescue cancer cells from anoxia-dependent cell death, and its inhibition may prevent important aspects of the adaptive stress response to hypoxia [17, 18]. Increasing evidence indicates that various types of cancer exhibit enhanced activation of the polyol pathway, as shown by elevated expression of enzymes of this pathway, most notably aldose reductase, and by polyol accumulation [19–22]. To our knowledge, we show for the first time the activation of the polyol pathway by hypoxia in human cancer cells. This is in line with a previous study, describing a similar phenomenon in rat PC12 neuronal cancer cells [17]. However, in contrast to PC12 cells, which trigger the polyol pathway under hypoxic conditions by up-regulating gene expression of aldose reductase [17], hypoxic human GBM cells seem to induce the polyol pathway through increased substrate availability (Fig. 1H) rather than by expression of aldose reductase (S3 Table), suggesting cell-type specific mechanisms involved in hypoxia-driven activation of the polyol pathway. The polyol pathway may confer resistance of glioma cells to hypoxia and drive cancer progression through pleiotropic mechanisms. Increased flux of glucose into the polyol pathway alters metabolism of cytosolic pyridine nucleotides to provide an increased ratio of NADP^+^/NADPH and NADH/NAD^+^ (Fig. 2A). As a consequence, increased NADP^+^/NADPH ratio sustains action of the pentose phosphate pathway (PPP), whereas an elevated NADH/NAD^+^ ratio may inhibit formation of 1,3-bisphosphoglycerate from glyceraldehyde-3-phosphate, resulting in glucose being converted into diacylglycerol (DAG) or pentoses through PPP [23]. These metabolic changes may thus supply the requirements of ribose 5-phosphate for DNA and RNA synthesis, NADPH for biosynthetic and antioxidant reactions as well as DAG for triacylglycerol and phospholipid synthesis. Activation of the polyol pathway may also contribute to proliferation under hypoxic conditions through the formation of fructose, which has been shown to induce transketolase activity in pancreatic cancer cells, driving fructose flux to the nonoxidative PPP and synthesis of nucleic acids [24]. Sorbitol derived from the polyol pathway may compete for intracellular stores of *myo*-inositol, inducing depletion of this metabolite [25]. As a consequence, modulated levels of *myo*-inositol may influence the biosynthesis and turnover of phospholipids. Furthermore, the osmotic property of polyols contributes to water and sodium retention, thereby regulating cell volume [26]. Thus, increased polyol levels could partially explain tumor associated oedema often observed in GBM; we found significantly increased sorbitol levels in conditioned medium at prolonged hypoxia (Fig. 2C). Finally, activation of the polyol pathway may be an indicator of increased metabolic flux through the aldose reductase pathway, which in addition to reducing glucose to sorbitol, catalyzes the reduction of a wide array of substances, including lipid aldehydes generated during lipid peroxidation and their glutathione-conjugates, phospholipids and steroids [26]. In this way, aldose reductase may be one of the cell’s defense mechanisms against hypoxic stress through multiple mechanisms, *e.g*. the activation of the polyol pathway, inactivation of highly reactive molecules and generation of inflammatory substances, triggering a pro-survival NFκB mediated signaling pathway.

An increased ratio of NADP^+^/NADPH and NADH/NAD^+^ at hypoxic conditions may lead to oxidative stress due to compromised reduction of glutathione. Further, an increased NADH/NAD^+^ ratio may block the activity of GAPDH and thus inhibit the formation of 1,3-bisphosphoglycerate from glyceraldehyde-3-phosphate, resulting in enhanced shunting into the pentose phosphate pathway (PPP), and diacylglycerol, triglyceride and phospholipid synthesis pathways (Fig. 2A and D). In support of this, we found that hypoxic GBM cells exhibit increased levels of glycolytic precursors of the PPP (Fig. 1H) as well as metabolic intermediates of the PPP, and the end product of this pathway, ribose 5-phosphate (Fig. 2E), *i.e*. a precursor of phosphoribosyl pyrophosphate (PRPP) that is required for the synthesis of purines and pyrimidines (S2 Fig.). However, despite elevated levels of ribose-5-phosphate and intermediates of the pyrimidine synthesis pathway, such as N-carbamoylaspartate and orotate (S2C Fig.), hypoxic GBM cells had decreased amounts of nucleotides and their metabolites, indicating that later steps of nucleotide synthesis were down-regulated (S2B and S2C Figs.).

As shown in Fig. 2F, activated nucleotide sugars are derived from glycolytic intermediates, *i.e*. glucose-6-phosphate and fructose-6-phosphate; hypoxia-driven induction of these metabolites (Fig. 1H) should result in increased levels of activated sugars. Contradicting this notion, hypoxic cells contained significantly less UDP-glucose (UDP-Glc) (Fig. 2G), possibly related to the recently demonstrated hypoxic induction of UDP-Glc utilization for glycogen synthesis [7, 27]. We found that hypoxic GBM cells express enhanced levels of several enzymes involved in glycogen metabolism (Fig. 2H). Interestingly, hypoxic cells showed increased levels of mRNA encoding UDP-Glc ceramide glucosyltransferase (UGCG) (Fig. 2I) that catalyzes the first glycosylation step in glycosphingolipid biosynthesis [28]; enhanced glycosylation of ceramides may contribute to the depletion of the intracellular UDP-Glc pool. Hypoxic cancer cells may also have an increased demand of UDP-galactose (UDP-Gal) and UDP-N-acetylglucosamine (UDP-GlcNAc)/UDP-N-acetylgalactosamine (UDP-GalNAc) for the synthesis of glycoproteins and glycolipids, as suggested by decreased UDP-Gal and UDP-GlcNAc/UDP-GalNAc (Fig. 2G), and increased levels of transcripts encoding beta-1,4-galactosyltransferase 1 (B4GalT1) and -4 (B4GalT4) (Fig. 2I). Further, we found that mannose-6-phosphate was induced by hypoxia in a time-dependent manner (up to approx. 3-fold) (Fig. 2F and G), and its conversion into GDP-fucose (GDP-Fuc) for glycosylation reactions was supported by increased GDP-Fuc (Fig. 2G) and a several-fold induction of fucosyltransferase 11 (FUT11) (Fig. 2I). In addition to hypoxia-induced expression of FUT11, we found increased mRNA levels of ST3 beta-galactoside alpha-2,3-sialyltransferase 6 (ST3Gal6) (Fig. 2I), which transfers sialic acid from the activated CMP-Neu5Ac to terminal positions of sugar chains on glycolipids (gangliosides) and to the N- or O-linked sugar chains of glycoproteins.

Together, these data indicate that hypoxia-induced import of glucose can modulate cellular glycosylation patterns and consequently GBM cell behavior in several ways. Indeed, glycolipids and glycoproteins emerge as interesting markers and targets of tumor-associated hypoxia [29]. For example, cancer cell adhesion to vascular endothelial cells during metastasis may be promoted through enhanced expression of genes encoding enzymes and transporters involved in the synthesis of sialyl Lewis x and sialyl Lewis a cell determinants [30]. The function of FUT11, which we found to be transiently up-regulated (> 5-fold) by hypoxia, remains unexplored; interestingly, a recent meta-analysis of genes differentially regulated in clear cell renal tumors, characterized by constitutive activation of HIFs, identified FUT11 as a top candidate biomarker of disease progression [31].

In addition to UDP-Glc, we observed a slight increase in the level of UDP-glucuronic acid (UDP-GlcA), one of the crucial substrates for glycosaminoglycan (GAG) synthesis, at 48 h of hypoxia (Fig. 2G). GAGs are long, unbranched polysaccharides that can reside in the glycocalyx or in the extracellular space as naked macromolecular saccharide chains, *i.e*. hyaluronic acid (HA), or conjugated to proteoglycans. These ECM constituents play critical roles in shaping the tumor microenvironment and GAGs have been found to be deregulated in cancer [32]. Increased UDP-GlcA levels suggest altered GAG synthesis at hypoxic conditions; however, the expression of the enzyme responsible for converting UDP-glucose to UDP-GlcA, UDP-glucose 6-dehydrogenase (UGDH), was not regulated by hypoxia in glioma cells (data not shown). The levels of sugar residues that together with GlcA make up the GAG chains, *i.e*. UDP-N-acetylglucosamine (UDP-GlcNAc) and UDP-N-acetylgalactosamine (UDP-GalNAc), were decreased at early time points and unchanged at 48 h of hypoxia, which may indicate a transient increase in their consumption. These divergent levels of specific UDP sugars at hypoxic conditions imply distinct glycosylation modifications in malignancies. Further supporting this notion, post-translational O-GlcNAcylation, *i.e*. the addition of a single GlcNac to serine or threonine residues of target proteins, has been shown to be elevated in various cancers [33]. O-GlcNAcylation is responsible for the posttranslational modification of numerous nuclear and cytosolic proteins including histone H2B thereby facilitating its monoubiquitination [34], and an open chromatin structure that is more accessible for transcription factors. It is tempting to speculate that O-GlcNAcylation would contribute to epigenetic modifications that constitute an alternative transcriptional control mechanism accompanying initial HIF-mediated transcription. Interestingly, an indirect O-GlcNAcylation-mediated mechanism of transcriptional control has been suggested where O-GlcNAc transferase increases HIF-1α levels by reducing the level of α-ketoglutarate, which is required for HIF-1α hydroxylation and subsequent proteasome degradation [35]. This may ultimately lead to the maintenance of glycolysis in cancer cells; hence, O-GlcNAcylation seems as an important post-translational modification in cancer cell metabolic reprograming.

### Hypoxia alters the metabolism of nucleotide cofactors NAD and NADP

**Fig 3:**
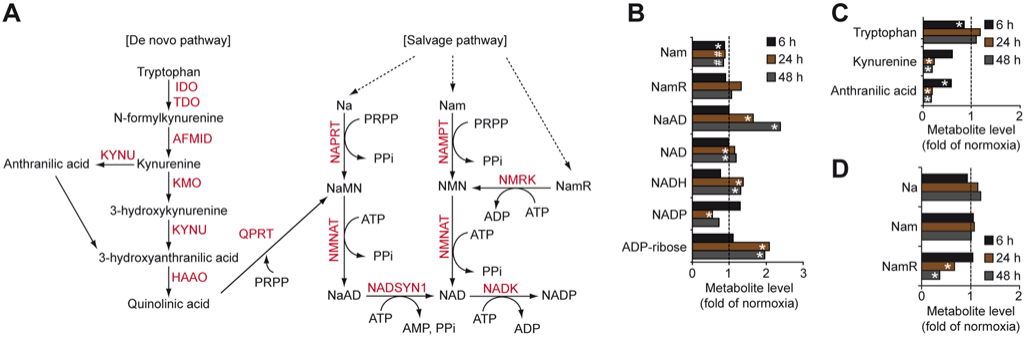
Hypoxic effects on the levels of nucleotide cofactors NAD and NADP. A, Shematic of metabolic pathways involved in the synthesis of nucleotide cofactors NAD and NADP. B-D, Hypoxic modulation of NAD, NADP and metabolites involved in their synthesis. Data represent fold change of metabolites levels in hypoxic GBM cells (B and C) and their conditioned media (D) compared with normoxic samples. * P < 0.05; # 0.05 < P < 0.1. HAAO, 3-hydroxyanthranilate 3,4-dioxygenase; IDO, indoleamine 2,3-dioxygenase; KMO, kynurenine 3-monooxygenase; KYNU, kynureninase; NADSYN1, NAD synthetase 1; NAMPT, nicotinamide phosphoribosyltransferase; NAPRT, nicotinate phosphoribosyltransferase; NMNAT, nicotinamide mononucleotide adenylyltransferase; NMRK, nicotinamide riboside kinase; QPRT, quinolinate phosphoribosyltransferase.

NAD is synthesized either in a *de novo* pathway from tryptophan or in salvage pathways from nicotinic acid (Na), nicotinamide (Nam) and nicotinamide riboside (NamR). NADP is synthesized from NAD in a reaction catalyzed by NAD kinase (NADK) (Fig. 3A) [36]. As depicted in Fig. 3B, hypoxic cells showed elevated levels of NAD, NADH and their precursor nicotinic acid dinucleotide (NaAD). NADP levels were decreased in hypoxic vs. normoxic cells (Fig. 3B), which may result from impaired phosphorylation of NAD as supported by diminished levels of NADK mRNA (S3 Table). In conjunction with increased levels of ribose-5-phosphate (Fig. 2E), and profound depletion of tryptophan metabolites, such as kynurenine and anthranilic acid (Fig. 3C), our data suggest enhanced *de novo* synthesis of NAD in hypoxic cells. Additionally, hypoxia may stimulate the synthesis of NAD in a salvage pathway from NamR as hypoxic cells consumed substantially more NamR from media than normoxic cells (Fig. 3D). Enhanced synthesis of NAD in hypoxic cells seems to be associated with profound utilization of NAD for the production of ADP-ribose (Fig. 3B), which can be further polymerized into poly-ADP-ribose, *i.e*. a key molecule of DNA repair mechanisms. Notably, NAD is also required for the action of the sirtuin enzymes (SIRT). The seven mammalian SIRTs are NAD-dependent lysine modifying deacetylases that act on proteins involved in various cancer related processes including proliferation, DNA damage repair, stress response and cell survival. Accordingly, high SIRT expression has been associated with a poor prognosis in many cancer types [37]. However, other studies point to a tumor suppressor role of SIRTs [38]. Interestingly, SIRT-mediated negative effects on tumor growth involve changes to various aspects of hypoxia regulated metabolism and even direct regulation of HIF1α. Accordingly, SIRT1 [39], SIRT3 [40, 41] and SIRT6 [42] have been reported to suppress HIF-1α resulting in repression of glycolysis, inhibition of mitochondrial ROS production and decreased expression of multiple glycolytic genes. The contradictory roles of SIRTs in human malignancies reflect the complex regulation of SIRT activities that depend on the tissue context, on distinct mechanisms of specific SIRT isoforms, the expression of SIRT substrates as well as on the access to NAD.

### Hypoxic GBM cells show down-regulation of the TCA cycle and profound accumulation of the oncometabolite 2-hydroxyglutarate

**Fig 4:**
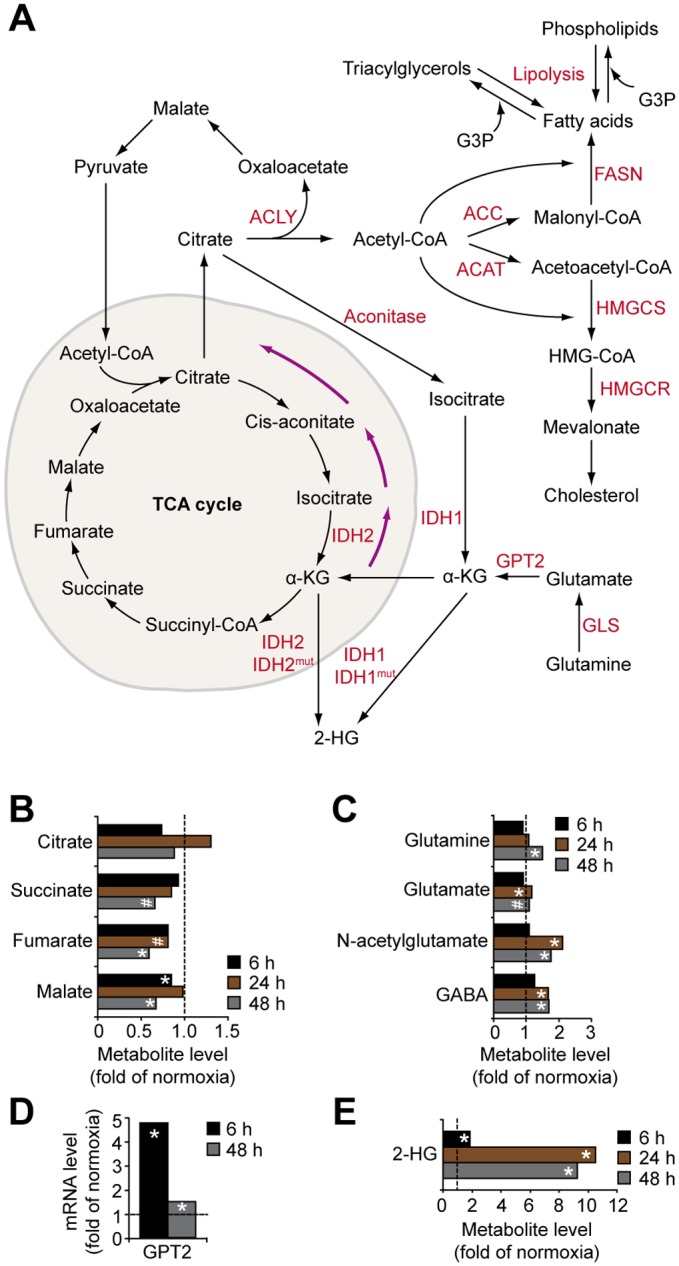
Hypoxic effects on TCA cycle and glutamine metabolism in GBM cells. A, Overview of TCA cycle and its role in lipid metabolism. B, Hypoxic modulation of TCA cycle metabolites in GBM cells. Data represent fold change of metabolite levels in hypoxic *vs*. normoxic GBM cells. C, Hypoxic modulation of glutamine and its metabolites in GBM cells. Data represent fold change of metabolite levels by hypoxia in GBM cells. D, Hypoxic induction of GPT2 mRNA; data represent fold change at hypoxia as compared with normoxia. E, Hypoxia dramatically increases 2-HG levels in GBM cells. * P < 0.05; # 0.05 < P < 0.1. ACAT, acetoacetyl-CoA thiolase; ACC, acetyl-CoA carboxylase; FASN, fatty acid synthase; GLS, glutaminase; HMGCS, 3-hydroxy-3-methylglutaryl-CoA synthase.

The breakdown of citrate by ATP-citrate lyase (ACLY) is a primary source of cytosolic acetyl-CoA that is used for protein acetylation as well as fatty acid and cholesterol synthesis [43–45] (Fig. 4A). Further, metabolism of cytosolic citrate by aconitase and isocitrate dehydrogenase (IDH) provides the cell with NADPH for redox regulation and synthesis of nucleotides, fatty acids and steroids. The generation of citrate involves the condensation of glutamine-derived oxaloacetate and glucose-derived acetyl-CoA. Our data suggest that hypoxia, especially at prolonged incubation, significantly decreases the levels of TCA cycle intermediates (Fig. 4B). The conversion of glucose-derived pyruvate to acetyl-CoA by PDH and of glutamine to oxaloacetate through the TCA cycle has been shown to be compromised under hypoxic conditions [46]. To support cell growth and viability, hypoxic cancer cells can reroute glutamine metabolism into the generation of citrate through reductive carboxylation of glutamine-derived α-ketoglutarate, a reaction mediated by the NADPH-dependent isoforms of IDH1 and IDH2 [46, 47] (Fig. 4A). Thus, stable levels of citrate through the course of hypoxia (Fig. 4B) may indicate that this metabolite was replenished via IDH-mediated reductive carboxylation of glutamine-derived α-ketoglutarate. In support of this idea, hypoxic GBM cells contained elevated levels of glutamine precursor and its derivatives (Fig. 4C), and increased expression (almost 5-fold *vs*. normoxic cells) of glutamic pyruvate transaminase 2 (GPT2) that catalyses the conversion of glutamate into α-ketoglutarate (Fig. 4A and D).

Interestingly, hypoxic GBM cells exhibited profoundly increased (up to 11-fold) levels of 2-hydroxyglutarate (2-HG) (Fig. 4E). Increased 2-HG may accompany the accumulation of glutamine-derived α-ketoglutarate and its increased reductive carboxylation, catalysed by IDH1 and IDH2 [46]. More importantly, 2-HG emerges as an oncometabolite during the induction of several types of cancer, including gliomas [47]. IDH1 and IDH2 are mutated in more than half of low-grade gliomas and secondary GBM, but more seldom in primary GBM (approx. 1/20), resulting in neo-enzymatic conversion of α-ketoglutarate to 2-HG. Although the exact role of IDH mutations in tumor development remains unclear, 2-HG may competitively inhibit α-ketoglutarate—dependent dioxygenase enzymes, *e.g*. prolyl hydroxylases, causing epigenetic alterations, abnormal collagen maturation, and down-regulation of HIF-dependent tumor suppressive signaling. A complex interplay between oncogenetic events (IDH mutations) and microenvironmental events (hypoxia) may thus be envisioned in glioma development with a dual role of 2-HG as a tumor initiator and as a suppressor of later stages of progressive disease.

### Hypoxia impairs cholesterol, glycerolipid and sphingolipid metabolism in GBM cells

**Fig 5:**
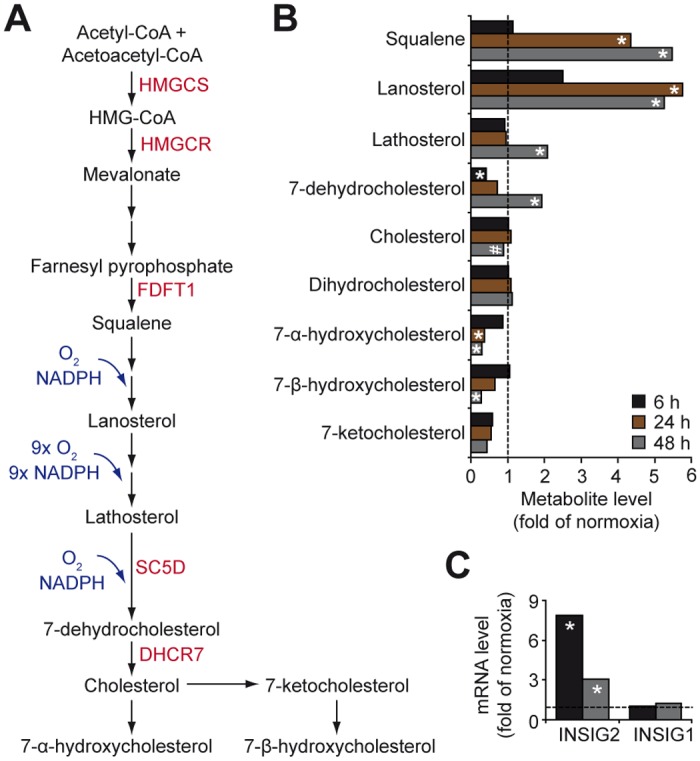
Hypoxic accumulation of cholesterol precursors in GBM cells. A, Schematic of cholesterol metabolism. B, Hypoxic modulation of metabolites involved in cholesterol metabolism, expressed as fold change in hypoxic compared with normoxic GBM cells. C, Hypoxic effects on INSIG-1 and -2 mRNAs in U87 MG cells. * P < 0.05; # 0.05 < P < 0.1. DHCR7, 7-dehydrocholesterol reductase; FDFT1, farnesyl-diphosphate farnesyltransferase 1; SC5D, sterol-C5-desaturase.

Cholesterol is a major membrane component and regulator of cell signaling and membrane transport by supporting the structure and function of lipid rafts, and is a precursor molecule for the biosynthesis of steroid hormones, vitamin D and bile acids [48]. Cholesterol level is regulated by *de novo* synthesis through the mevalonate pathway, where conversion of 3-hydroxy-3-methylglutaryl CoA (HMG-CoA) to mevalonate by HMG-CoA reductase (HMGCR) constitutes the rate-limiting step, and by uptake of cholesterol-enriched lipoproteins [48]. The reductase is subjected to feedback control by sterols and nonsterol end-products of mevalonate metabolism [49], partly through proteasomal degradation of HMGCR by insulin-induced gene (Insig) proteins in the endoplasmic reticulum [50]. We found that hypoxic cells display profound accumulation of squalene, lanosterol and lathosterol, whose conversion into cholesterol requires oxygen (Fig. 5A and B). Moreover, hypoxic cells showed increased expression (approx. 8-fold) of Insig2 (Fig. 5C). Given that lanosterol serves as a signal for Insig-dependent ubiquitination and degradation of HMGCR this represents another potential mechanism of hypoxia-mediated down-regulation of cholesterol synthesis [50, 51]. Intriguingly, however, hypoxia did not result in decreased total cholesterol in GBM cells (Fig. 5B), suggesting compensatory mechanisms to maintain cholesterol levels. GBM cells contained reduced amounts of 7-α- and 7-β-hydroxycholesterol (Fig. 5B), suggesting decreased conversion of cholesterol into oxysterols as one potential mechanism. Additional possibilities that remain to be investigated are hypoxic induction of cholesterol uptake [52], or stimulation of its release from lipid droplets.

**Fig 6:**
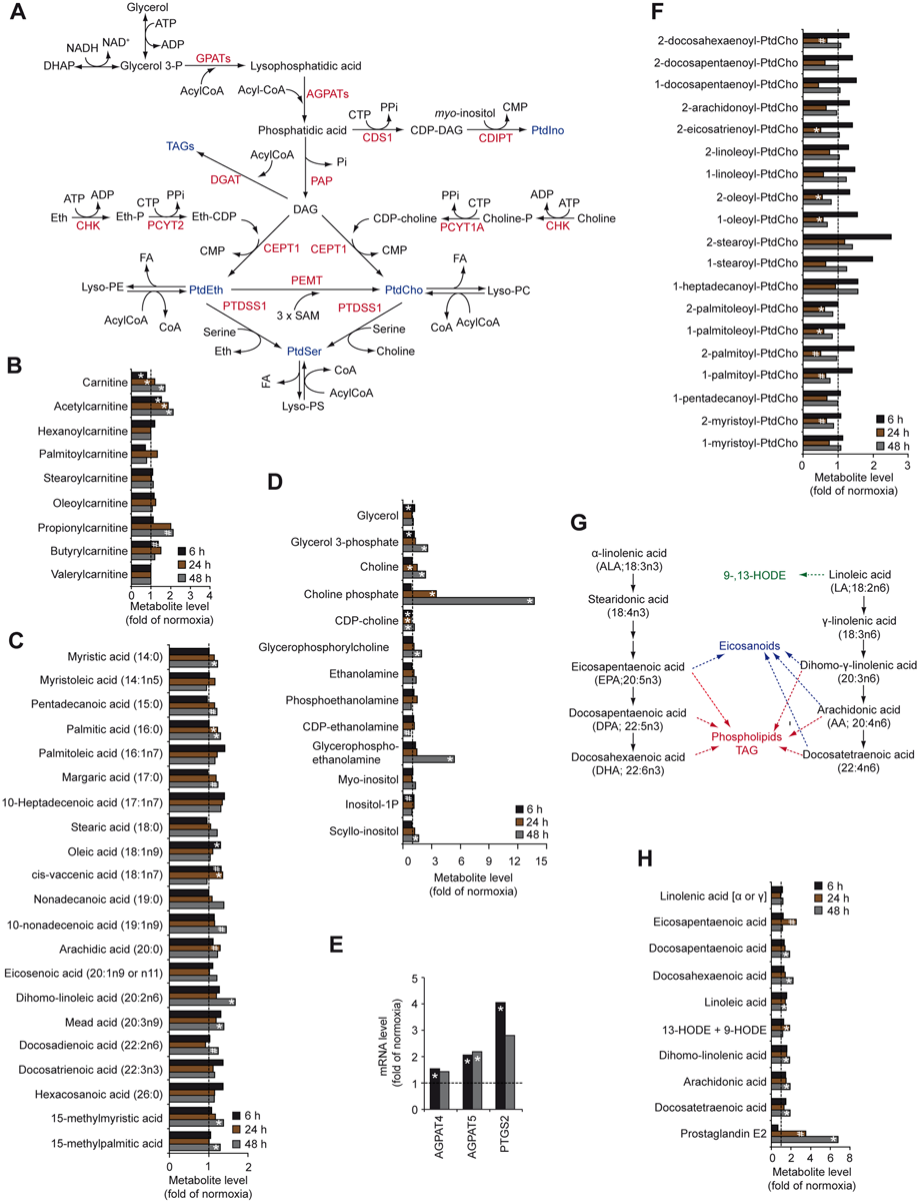
Effects of hypoxia on glycerolipid metabolism in GBM cells. A, Schematic illustration of glycerolipid metabolism. B-D, Hypoxic modulation of metabolites involved in fatty acid β-oxidation (B), fatty acids (C), and metabolites involved in the synthesis of triacylglycerols and phospholipids (D) in GBM cells. E, Hypoxic induction of mRNAs of enzymes involved in glycerolipid metabolism at the indicated time-points. Data represent fold change in hypoxic as compared with normoxic cells. F, Effects of hypoxia on phosphatidylcholine-derived lysolipids in GBM cells expressed as fold change in hypoxic compared with normoxic cells. G, Schematic graph of essential fatty acid metabolism. H, Hypoxic regulation of essential fatty acids and their metabolites in GBM cells. * P < 0.05; # 0.05 < P < 0.1. CDIPT, CDP-diacylglycerol-inositol 3-phosphatidyltransferase; CDS1, CDP-diacylglycerol synthase 1; CEPT1, choline/ethanolamine phosphotransferase 1; CHK, choline kinase; DGAT, diglyceride acyltransferase; PAP, phosphatidate phosphatase; PCYT1A, choline-phosphate cytidylyltransferase 1A; PCYT2, ethanolamine-phosphate cytidylyltransferase 2; PEMT, phosphatidylethanolamine N-methyltransferase; PTDSS1, phosphatidylserine synthase 1.

Polar glycerophospholipids, including phosphatidylethanolamine (PtdEth), phosphatidylserine (PtdSer), phoshatidylcholine (PtdCho) and phosphatidylinositol (PtdIno) serve as structural components of cellular membranes and second messengers in signal transduction. *De novo* synthesis of glycerolipids begins with acylation of glycerol-3-phosphate by glycerol-3-phosphate acyltransferases (GPATs), generating lysophosphatidic acid, which is subsequently acylated to phosphatidic acid by 1-acylglycerol-3-phosphate acyltransferases (AGPATs). Phosphatidic acid can then be converted into phosphatidic acid, a precursor of PtdIno, or metabolized by phosphatidate phosphatase (PAP) to CDP-diacylglycerol (DAG), a precursor of PtdCho, PtdEth, PtdSer and triacylglycerols (TAGs) (Fig. 6A). Our data suggest that hypoxia does not seem to deplete metabolites of fatty acid β-oxidation (Fig. 6B), but appeared to increase the levels of palmitic acid, glycerol-3-phosphate, choline and choline phosphate (Fig. 6C and D). Abnormal choline metabolism has been associated with tumor initiation and progression in glioma and several other tumor types [53], and may provide a non-invasive biomarker of glioma transformation and response to therapy.

Together with gene expression analysis showing hypoxic induction of AGPAT4 and AGPAT5, *i.e*. enzymes involved in the synthesis of phosphatidic acid (Fig. 6E), our data suggest that hypoxic GBM cells exhibit increased synthesis of TAGs and PtdCho. Hypoxic cells also contained decreased levels of lysophospholipids mostly derived from PtdCho and PtdEtn but not from PtdSer and PtdIno (Fig. 6F and S3 Fig.). This effect could result from lipase-driven degradation of lysophospholipids into glycerolphosphorylcholine and glycerophosphorylethanolamine (Fig. 6D), which may further explain elevated levels of α-linolenic acid-derived, polyunsaturated omega-3 fatty acids, and linoleic acid-derived, polyunsaturated omega-6 fatty acids, in hypoxic *vs*. normoxic cells (Fig. 6G and H). Additionally, we found hypoxia-induced conversion of linoleic acid to 13-hydroxyoctadecadienoic acid (13-HODE) and 9-HODE as well as arachidonic acid to prostaglandin E2 in GBM cells (Fig. 6G and H). Accordingly, hypoxic GBM cells displayed transcriptional activation of prostaglandin-endoperoxide synthase 2 (PTGS2, also known as cyclooxygenase-2, COX-2) that catalyzes the conversion of arachidonic acid to prostaglandin E2 (Fig. 6E). COX-2 expression was previously shown to be increased in high as compared with low grade human gliomas and correlated with worse patient outcome [54]. Mechanistically, this may be explained by arachidonic acid-mediated stimulation of GBM cell migration and infiltration, a process dependent on association with the brain fatty acid binding protein (FABP7) [55]; FABP7 was significantly up-regulated (approx. 1.6-fold, P<0.05) by hypoxia in GBM cells. COX-2 inhibitors, mainly celecoxib, emerge as interesting cancer preventive agents, but also may sensitize GBM cells to radiotherapy by augmenting ER stress [56]. Another interesting possibility, supported by recent *in vivo* studies, is efficient attenuation of the immunosuppressive function of prostaglandin E2 by intratumoral administration of COX-2 inhibitors [57].

Sphingolipids are structural components of cellular membranes and important mediators in cellular signaling events [58]. *De novo* sphingolipid synthesis involves the conversion of palmitoyl-CoA and serine into ceramide (S4A Fig.). Ceramide may be glycosylated by glucosylceramide synthase, yielding glycosphingolipids, or be converted into sphingomyelin by the action of sphingomyelin synthase that in the reverse reaction may be broken down by sphingomyelinase to form ceramide. Breakdown of ceramide by ceramidase yields sphingosine, which upon phosphorylation forms sphingosine-1-phosphate (S4A Fig.). We found that hypoxic cells had decreased levels of sphinganine and 2-hydroxy fatty acids (S4B and S4C Fig.), suggesting that *de nov*o synthesis of pro-apoptotic ceramides is down-regulated. These observations, together with increased transcriptional activation of UGCG (Fig. 2I) that mediates the formation of glucoceramides, indicate that GBM cells may adapt to hypoxic stress by decreasing the pool of intracellular ceramides and increasing their conversion into anti-apoptotic glycosphingolipids. Another interesting possibility is cellular ceramide metabolite detoxification through increased release of exosomes [59]. Indeed, cancer cell release of microvesicles was shown to be induced by hypoxia in a HIF-dependent manner [60]. Further, exosomes isolated from GBM patients were shown to closely reflect the signalling status of hypoxic donor cells [61]. The role of exosomes as transporters of sphingolipids in the hypoxic tumor microenvironment clearly deserves further investigation.

### Enhanced catabolism of proteins and amino acids in hypoxic GBM cells

**Fig 7:**
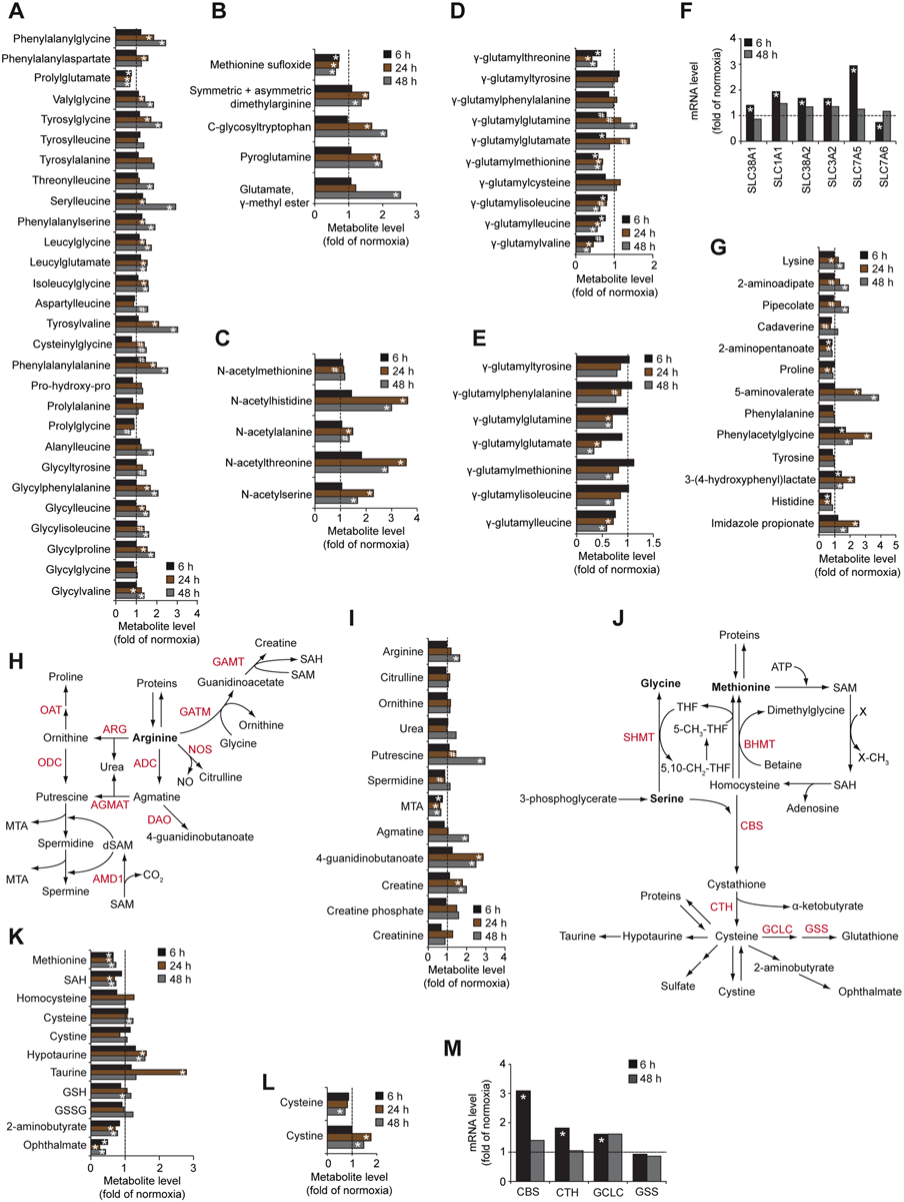
Protein and amino acid metabolism in hypoxic GBM cells. A-C, Hypoxic regulation of dipeptides (A), post-translationally modified amino acids (B) and acetylated amino acids (C) expressed as fold change in hypoxic *vs*. normoxic cells. D and E, Effects of hypoxia on the gamma-glutamyl cycle in GBM cells (D) and their conditioned media (E) compared with normoxic samples. F, Hypoxic effects on transcriptional activation of amino acid transporters expressed as fold change of mRNA levels at hypoxia *vs*. normoxia. G, Hypoxic modulation of amino acid catabolism in GBM cells. H, Schematic of arginine metabolism. I, Effects of hypoxia on arginine and related metabolites. J, Schematic of methionine and serine metabolism. K and L, Hypoxic modulation of methionine and its metabolites in GBM cells (K) and their conditioned media (L). M, Hypoxic effects on mRNAs of proteins involved in cysteine and glutathione synthesis. * P < 0.05; # 0.05 < P < 0.1. ADC, arginine decarboxylase; AGMAT, agmatinase; AMD1, adenosylmethionine decarboxylase 1; ARG, arginase; BHMT, betaine-homocysteine methyltransferase; CBS, cystathionine beta synthase; CTH, cystathionine gamma-lyase; DAO, diamine oxidase; GAMT, guanidinoacetate N-methyltransferase; GATM, glycine amidinotransferase; GCLC, glutamate-cysteine ligase; GSS, glutathione synthetase; NOS, nitric oxide synthase; OAT, ornithine aminotransferase; ODC, ornithine decarboxylase; SHMT, serine hydroxymethyltransferase.

Prolonged hypoxia resulted in the accumulation of dipeptides and amino acids with various posttranslational modifications (Fig. 7A-C), suggesting that hypoxic GBM cells increase their pool of free amino acids to fulfill the energetic demands that cannot be sufficiently provided from the glycolytic flux. Hypoxia decreased the levels of numerous γ-glutamyl-amino acids both in cells (Fig. 7D) and in conditioned media (Fig. 7E). As anti-oxidant glutathione provides the γ-glutamyl moiety in the γ-glutamyl cycle, GBM cells may decrease the activity of this pathway to limit glutathione consumption under hypoxic conditions. Increased expression of genes encoding various amino acids transporters (Fig. 7F), indicate that hypoxic GBM cells may be more dependent on this amino acid uptake pathway rather than on the γ-glutamyl cycle. Notably, both SLC3A2 and SLC7A5, that together heterodimerize into the large neutral amino acid transporter (LAT1 or CD98) were significantly up-regulated by hypoxia. High LAT1 expression has been shown to correlate with poor survival in GBM patients, and specific inhibition of LAT1 was shown to attenuate glioma growth [62].

Hypoxic GBM cells exhibited increased levels of *e.g*. lysine-derived 2-aminoadipate and pipecolate, proline-derived 5-aminovalerate, phenylalanine-derived phenylacetylglycine, tyrosine-derived 3-(4-hydroxyphenyl)lactate and histidine-derived imidazole propionate (Fig. 7G), suggesting increased catabolism of proteins. These findings are consistent with enhanced autophagy and protein degradation by chronic hypoxia [27]. Indeed, we found that several autophagy-related transcripts were significantly induced by hypoxia in GBM cells (*e.g*. BNIP3L: 4.5-fold; ULK1: 2.4-fold; ATG9: 1.8-fold; VPS34: 1.7-fold as compared with normoxia). Further, hypoxia-driven degradation of arginine could result in elevated levels of the growth promoting polyamine putrescine (Fig. 7H and I) that was previously shown to accumulate in hypoxic cancer cells and to protect them from hypoxic stress [63].

Finally, hypoxia enhanced cellular levels of cysteine-derived antioxidant metabolites, such as glutathione and taurine (Fig. 7J and K) with a corresponding increased consumption of extracellular cysteine (Fig. 7L). Transcriptional activation of enzymes involved in cystathionine-beta-synthase (CBS), cystathionine gamma-lyase (CTH), and glutamate-cysteine ligase (GCLC) (Fig. 7M), may support increased synthesis of cysteine and glutathione in hypoxic GBM cells.

## Conclusions

It is well-established that hypoxia and down-stream features of the tumor microenvironment, *e.g*. acidosis correlate with GBM tumor aggressiveness and patient outcome. This gives strong rationale for studies aiming at the identification of hypoxia-induced metabolic alterations with the potential to provide a biochemical basis for the development of therapeutic strategies that specifically kill the most aggressive tumor cells. Also, further development and clinical implementation of magnetic resonance and positron emission tomography imaging techniques for non-invasive *in vivo* analysis of the metabolic status of relatively inaccessible brain tumor lesions would clearly benefit from such studies. Importantly, several low-molecular weight metabolites and amino acids readily visible in proton MR spectra were significantly and time-dependently increased by hypoxia in GBM cells (*e.g*. choline, phosphocholine, glycerophosphocholine, creatine, taurine, lysine, glutamate/glutamine, and polyamine; see Figs. 6 and 7). This may, at least partially, explain the utility of this technique to distinguish between low and high grade glioma tumors [64].

As opposed to molecular profiling of clinical specimens that is limited by tumor heterogeneity, steady-state measurements and the potential influence of different handling of tissue post resection, cell culture models provide a powerful tool for dynamic studies at controlled, microenvironmental conditions. Indeed, we found several metabolites to be transiently regulated by hypoxia, *e.g*. glucose, fructose, lysophospholipids, taurine, and orotate, although most metabolic changes were found to increase with prolonged hypoxic stress. Chinnayian *et al*. recently performed a global metabolomic analysis of glioma tumors, proposing a biochemical profile that may differentiate low- from high-grade tumors [65]. Notably, their finding of accumulation of glucose-6-P and key metabolites of the PPP (6-phosphogluconate and ribose-5-P) as a major discriminator between GBM and low-grade gliomas corresponds with our data, showing a highly significant and time-dependent increase of these metabolites in hypoxic GBM cells (Fig. 1 and 2). This metabolic phenotype reflects shunting of glycolytic carbon into macromolecular biosynthesis, and as an important consequence, increased generation of reducing potential to counteract the toxic effects of oxidative species. This notion was supported by enhanced level of reduced glutathione in high-grade *vs*. low grade glioma [65] as well as in hypoxic *vs*. normoxic GBM cells (present study; Fig. 7).

Our data support the notion that gene expression data can provide important clues to and in many cases is in good agreement with metabolic profiling. HIFs and other oxygen-regulated genes operate to genetically adapt cancer cells to hypoxic stress; metabolic analyses, including our study, point at a complex cross-talk between genetic and biochemical changes where metabolites, *e.g*. 2-HG, contribute to the metabolic status of hypoxic cells through regulation of HIF stability and by epigenetic events.

To conclude, our studies illustrate the power of combined transcriptomic analysis and comprehensive metabolomics for defining adaptive pathways of hypoxia in cancer cells. These new insights may be further exploited for the identification of treatment targets of hypoxic and highly aggressive tumors.

## Supporting Information

S1 Fig Glycolysis provides precursors for the synthesis of essential macromolecules. (TIF)

S2 Fig Effect of hypoxia on the metabolism of purines and pyrimidines. A, Schematic illustration of the purine (upper panel) and pyrimidine (lower panel) metabolism. B and C, Hypoxic modulation of metabolites involved in the purine (B) and pyrimidine metabolism (C). Data represent fold change of metabolite levels in hypoxic *vs*. normoxic cells. * P < 0.05; # 0.05 < P < 0.1. ADSL, adenylosuccinate lyase; ADSS, adenylosuccinate synthase; AFMID, arylformamidase; CAD, carbamoyl-phosphate synthetase 2; GMPS, guanine monphosphate synthetase; IMPDH, inosine-5′-monophosphate dehydrogenase; PRPS, phosphoribosyl pyrophosphate synthetase; UMPS, uridine monophosphate synthetase.
(TIF)

S3 Fig Hypoxia-driven effects on lysolipids derived from PtdEth, PtdIno and PtdSer. Data represent fold change of lysolipid levels in hypoxic *vs*. normoxic GBM cells. * P < 0.05.
(TIF)

S4 Fig Effects of hypoxia on sphingolipid metabolism in GBM cells. A, Illustration of sphingolipid metabolism. B and C, Hypoxic effects on metabolites involved in sphingolipid metabolism. Data represent fold change of metabolite levels in hypoxic GBM cells *vs*. normoxic samples. * P < 0.05; # 0.05 < P < 0.1. CDase, ceramidase; CERS, ceramide synthase; DEGS, dihydroceramide desaturase; KDSR, 3-ketodihydrosphingosine reductase; SMase, sphingomyelinase; SMS, sphinogomyelin synthase; SPHK, sphingosine kinase; SPTLC, serine C-palmitoyltransferase; UGCG, UDP-glucose ceramide glucosyltransferase.
(TIF)

S1 Table List of identified metabolites in GBM cells with fold changes, p-values and q-values for comparison between normoxic and hypoxic samples. (XLS)

S2 Table List of identified metabolites in conditioned media from GBM cells with fold changes, p-values and q-values for comparison between normoxic and hypoxic samples. (XLS)

S3 Table Hypoxia-modulated mRNAs encoding for proteins involved in U87 MG cell metabolism, as determined by Illumina gene expression analysis. (XLS)
