# Supplementary figures and images for: Global Profiling of Metabolic Adaptation to Hypoxic Stress in Human Glioblastoma Cells
Source: PLoS One. 2015 Jan 29;10(1):e0116740. doi: 10.1371/journal.pone.0116740 (PMC4310608; doi:10.1371/journal.pone.0116740)

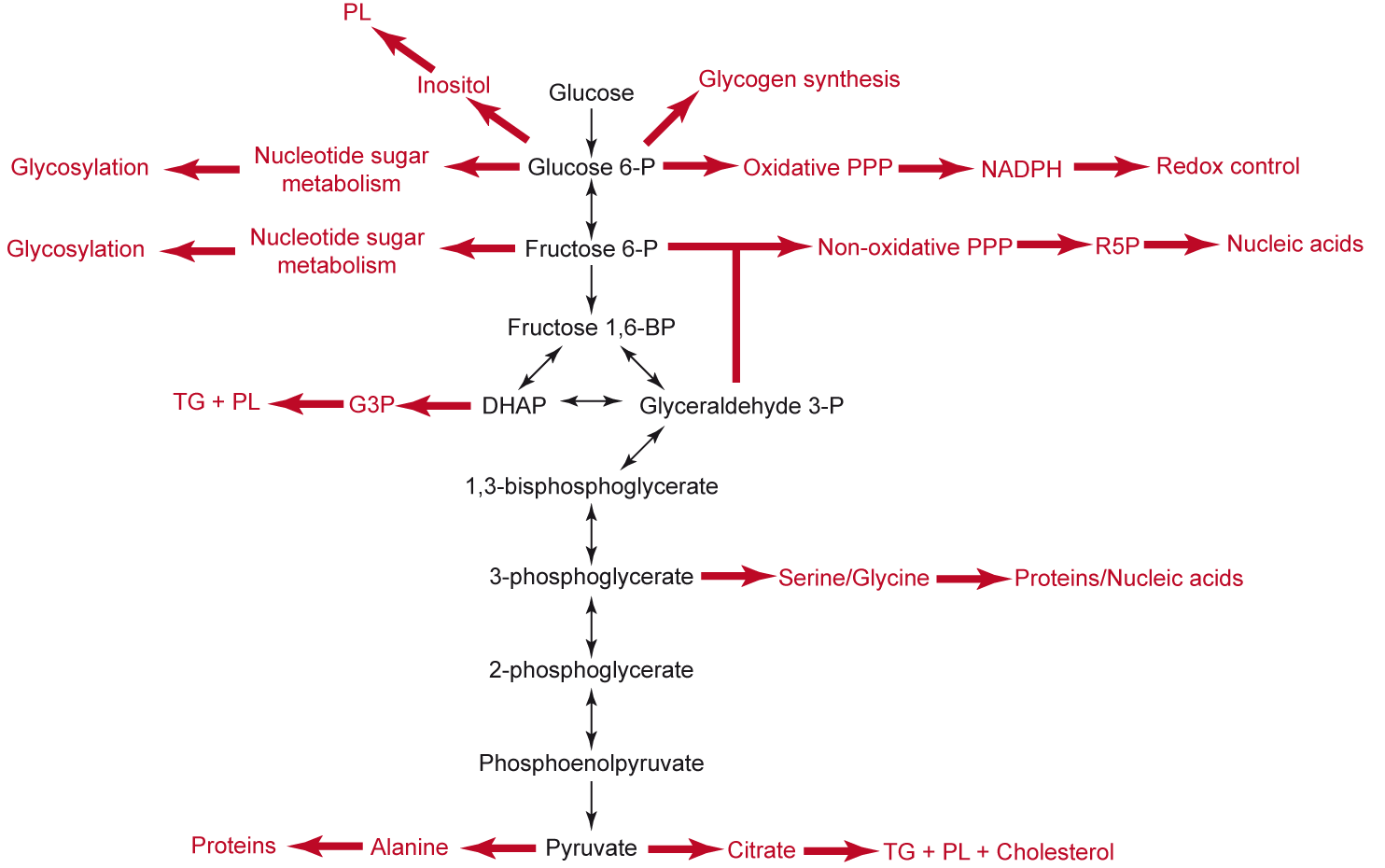

Supplement: S1 Fig — (TIF) [file pone.0116740.s001.tif]

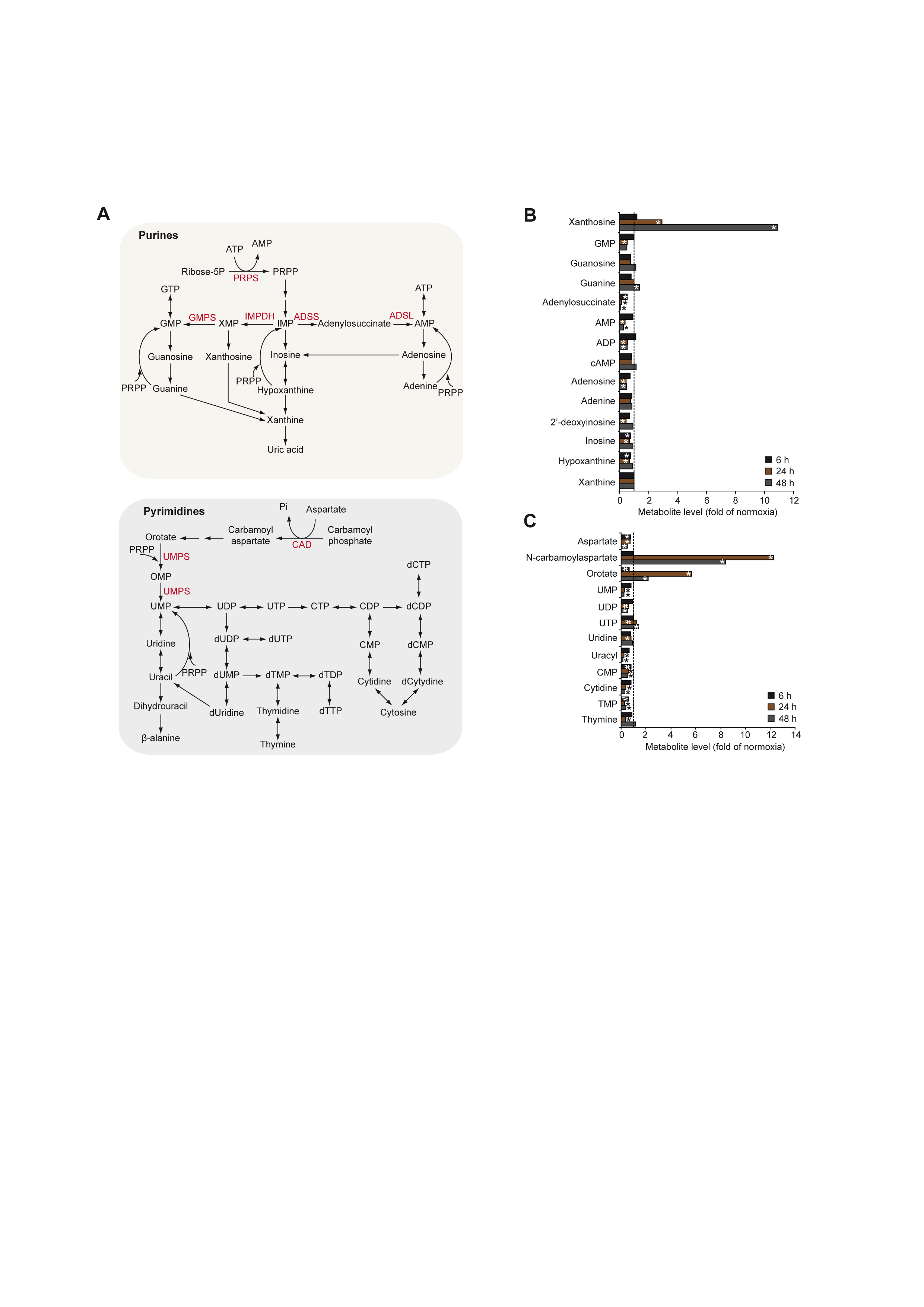

Supplement: S2 Fig — A, Schematic illustration of the purine (upper panel) and pyrimidine (lower panel) metabolism. B and C, Hypoxic modulation of metabolites involved in the purine (B) and pyrimidine metabolism (C). Data represent fold change of metabolite levels in hypoxic vs. normoxic cells. * P < 0.05; # 0.05 < P < 0.1. ADSL, adenylosuccinate lyase; ADSS, adenylosuccinate synthase; AFMID, arylformamidase; CAD, carbamoyl-phosphate synthetase 2; GMPS, guanine monphosphate synthetase; IMPDH, inosine-5′-monophosphate dehydrogenase; PRPS, phosphoribosyl pyrophosphate synthetase; UMPS, uridine monophosphate synthetase. (TIF) [file pone.0116740.s002.tif]

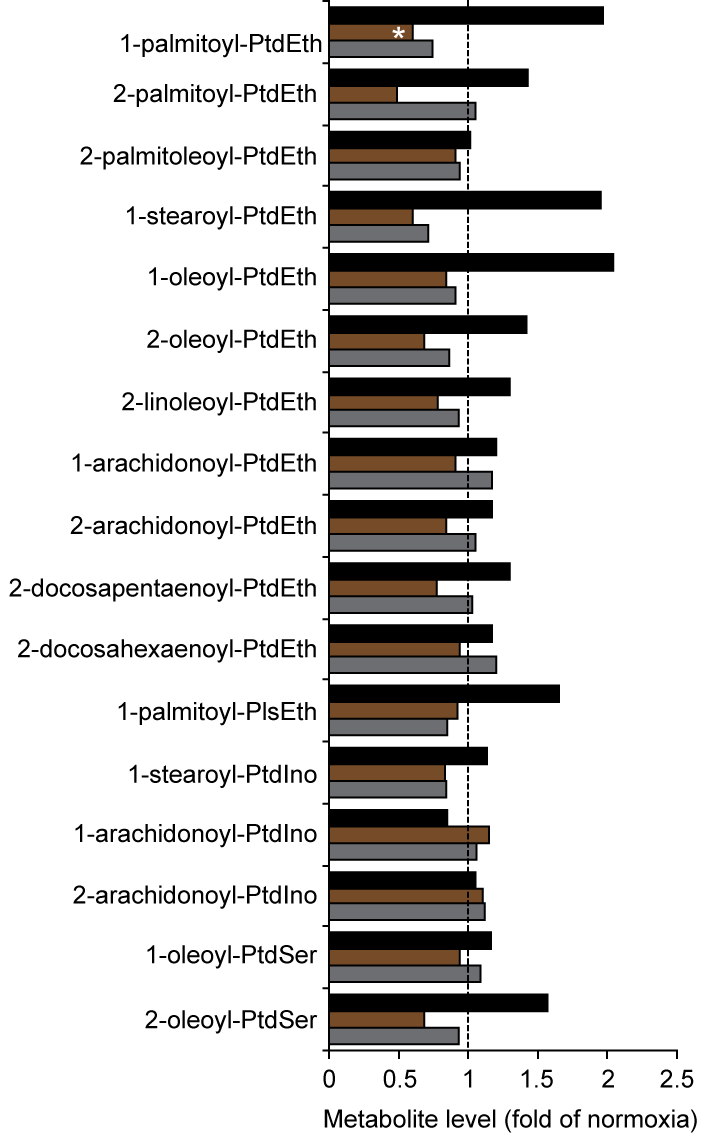

Supplement: S3 Fig — Data represent fold change of lysolipid levels in hypoxic vs. normoxic GBM cells. * P < 0.05. (TIF) [file pone.0116740.s003.tif]

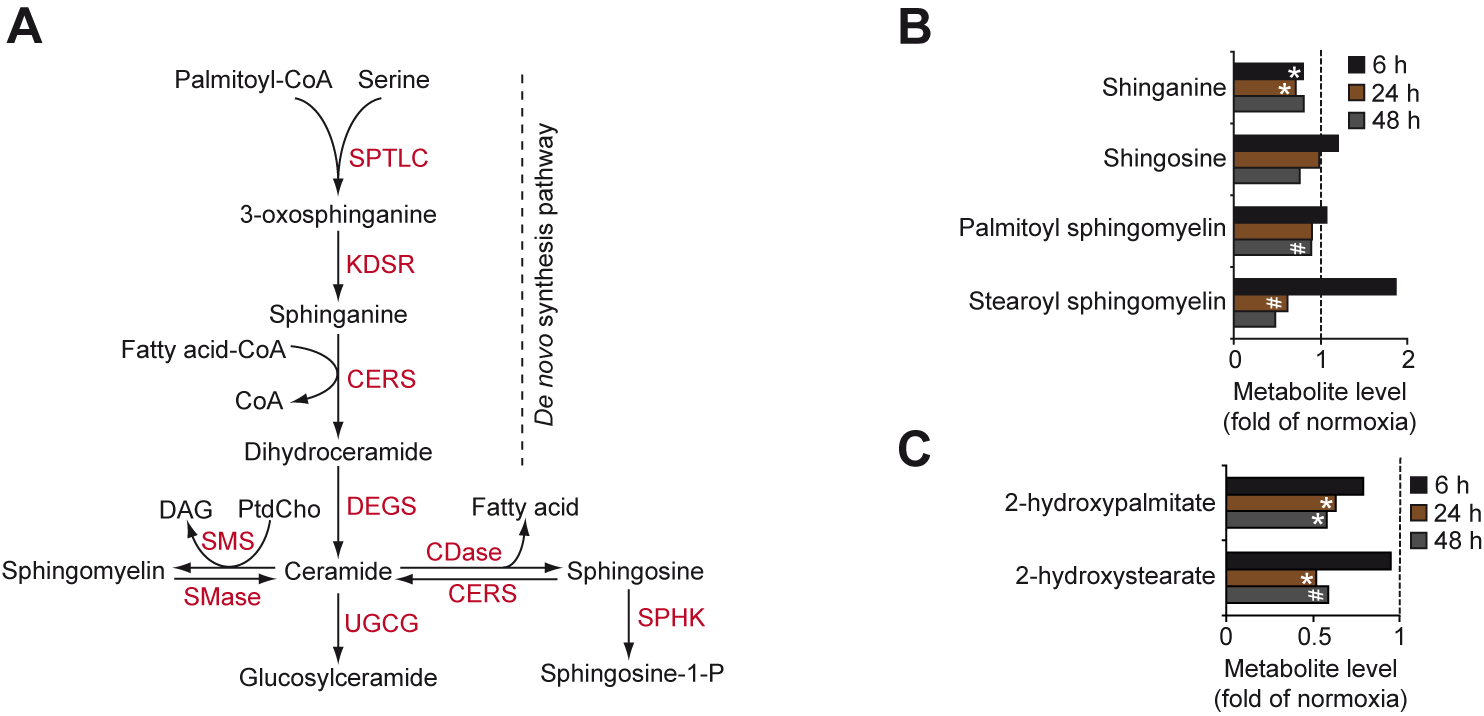

Supplement: S4 Fig — A, Illustration of sphingolipid metabolism. B and C, Hypoxic effects on metabolites involved in sphingolipid metabolism. Data represent fold change of metabolite levels in hypoxic GBM cells vs. normoxic samples. * P < 0.05; # 0.05 < P < 0.1. CDase, ceramidase; CERS, ceramide synthase; DEGS, dihydroceramide desaturase; KDSR, 3-ketodihydrosphingosine reductase; SMase, sphingomyelinase; SMS, sphinogomyelin synthase; SPHK, sphingosine kinase; SPTLC, serine C-palmitoyltransferase; UGCG, UDP-glucose ceramide glucosyltransferase. (TIF) [file pone.0116740.s004.tif]
